# Supplementary figures and images for: UBR-1 deficiency leads to ivermectin resistance in Caenorhabditis elegans
Source: eLife. 2025 Apr 1;13:RP103718. doi: 10.7554/eLife.103718 (PMC11961118; doi:10.7554/eLife.103718)

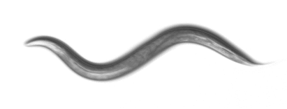

Supplement: Source data 1. [file elife-103718-data1.zip › Figure 1-source data/Figure 1-source data 4(E)/ubr-1 (IVM=0).png]

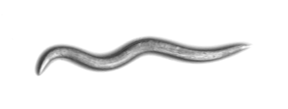

Supplement: Source data 1. [file elife-103718-data1.zip › Figure 1-source data/Figure 1-source data 4(E)/ubr-1 (IVM=5).png]

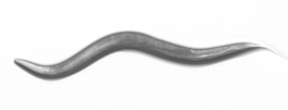

Supplement: Source data 1. [file elife-103718-data1.zip › Figure 1-source data/Figure 1-source data 4(E)/WT (IVM=0).png]

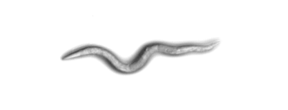

Supplement: Source data 1. [file elife-103718-data1.zip › Figure 1-source data/Figure 1-source data 4(E)/WT (IVM=5).png]

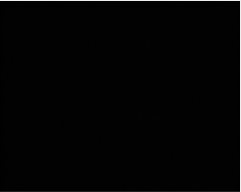

Supplement: Source data 1. [file elife-103718-data1.zip › Figure 2-figure supplement 1-source data/Figure 2-figure supplement 1-source data1(A)/+Cef.png]

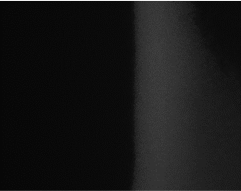

Supplement: Source data 1. [file elife-103718-data1.zip › Figure 2-figure supplement 1-source data/Figure 2-figure supplement 1-source data1(A)/Condensed+Cef.png]

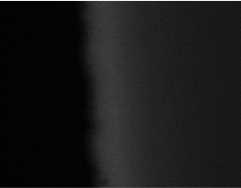

Supplement: Source data 1. [file elife-103718-data1.zip › Figure 2-figure supplement 1-source data/Figure 2-figure supplement 1-source data1(A)/Control.png]

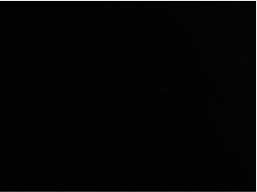

Supplement: Source data 1. [file elife-103718-data1.zip › Figure 2-figure supplement 1-source data/Figure 2-figure supplement 1-source data1(A)/Diluted.png]

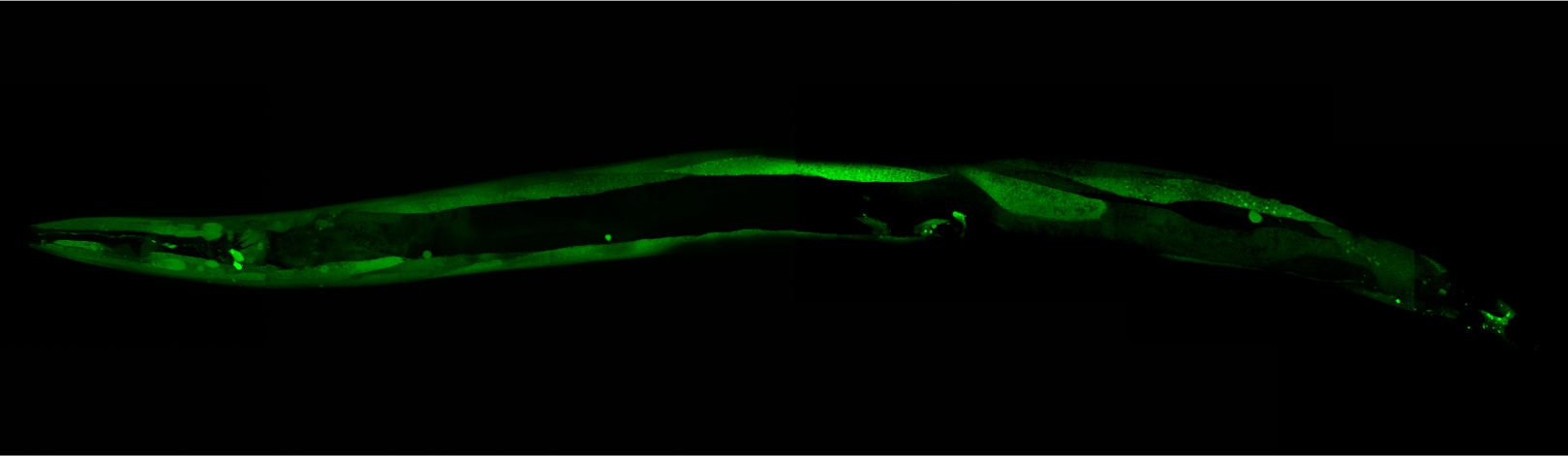

Supplement: Source data 1. [file elife-103718-data1.zip › Figure 3-figure supplement 1-source data/Figure 3-figure supplement 1-source data1(A)/UBR-1 expression.png]

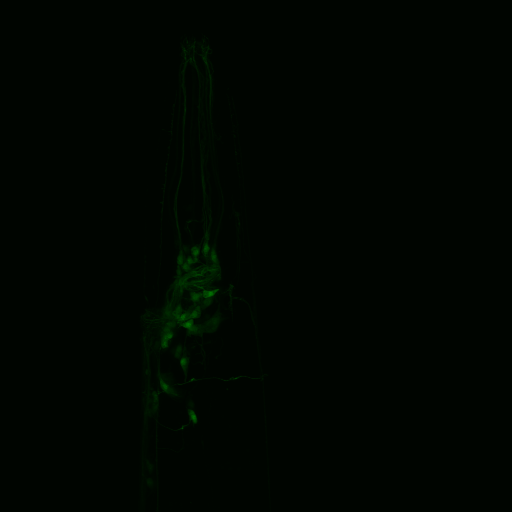

Supplement: Source data 1. [file elife-103718-data1.zip › Figure 4-figure supplement 1-source data/Figure 4-figure supplement 1-source data1(A)/ubr-1-AVR-14-GFP.png]

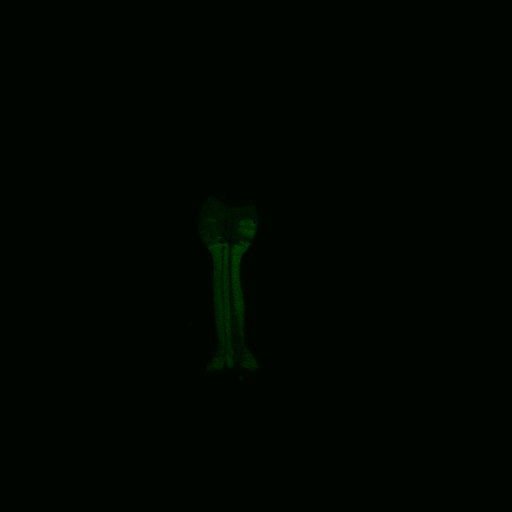

Supplement: Source data 1. [file elife-103718-data1.zip › Figure 4-figure supplement 1-source data/Figure 4-figure supplement 1-source data1(A)/ubr-1-AVR-15-GFP.png]

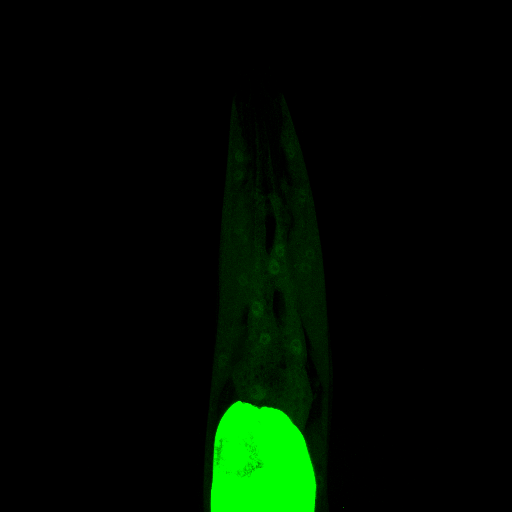

Supplement: Source data 1. [file elife-103718-data1.zip › Figure 4-figure supplement 1-source data/Figure 4-figure supplement 1-source data1(A)/ubr-1-GLC-1-GFP.png]

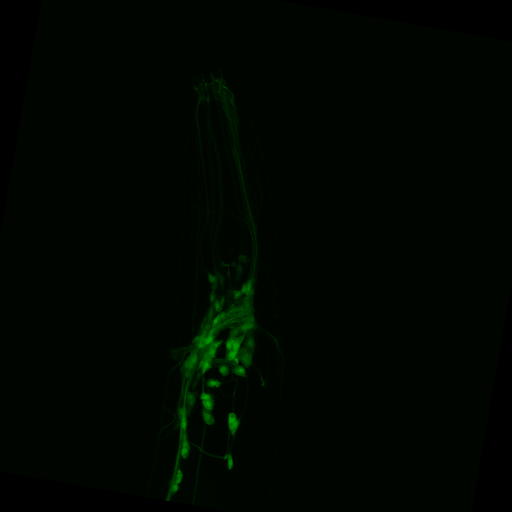

Supplement: Source data 1. [file elife-103718-data1.zip › Figure 4-figure supplement 1-source data/Figure 4-figure supplement 1-source data1(A)/WT-AVR-14-GFP.png]

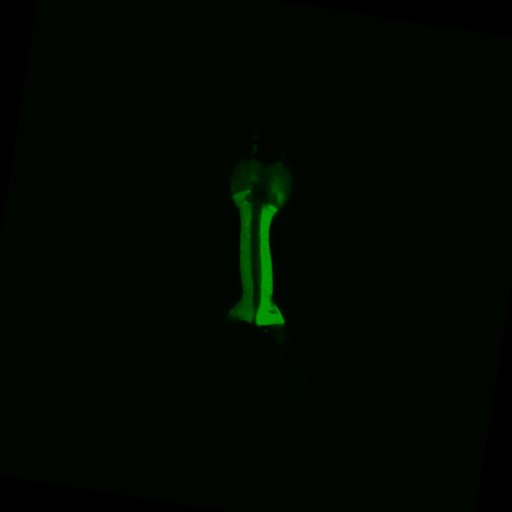

Supplement: Source data 1. [file elife-103718-data1.zip › Figure 4-figure supplement 1-source data/Figure 4-figure supplement 1-source data1(A)/WT-AVR-15-GFP.png]

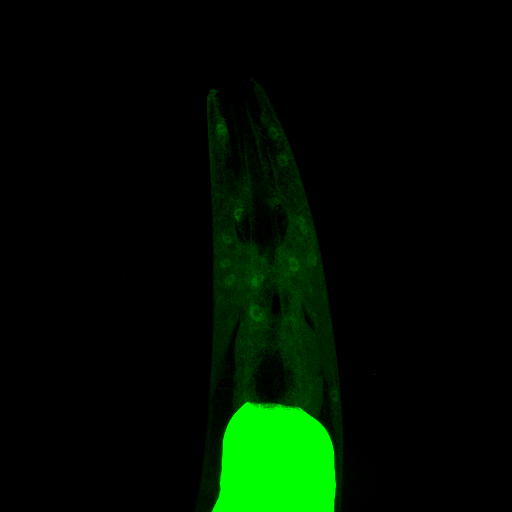

Supplement: Source data 1. [file elife-103718-data1.zip › Figure 4-figure supplement 1-source data/Figure 4-figure supplement 1-source data1(A)/WT-GLC-1-GFP.png]

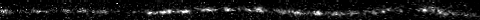

Supplement: Source data 1. [file elife-103718-data1.zip › Figure 4-figure supplement 2-source data/Figure 4-figure supplement 2-source data1(A)/ubr-1-unc-29-RFP.png]

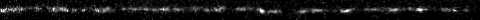

Supplement: Source data 1. [file elife-103718-data1.zip › Figure 4-figure supplement 2-source data/Figure 4-figure supplement 2-source data1(A)/wild type-unc-29-RFP.png]

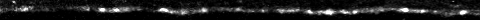

Supplement: Source data 1. [file elife-103718-data1.zip › Figure 4-figure supplement 2-source data/Figure 4-figure supplement 2-source data4(D)/ubr-1-UNC-49-GFP.png]

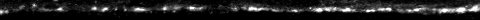

Supplement: Source data 1. [file elife-103718-data1.zip › Figure 4-figure supplement 2-source data/Figure 4-figure supplement 2-source data4(D)/wild type-UNC-49-GFP.png]

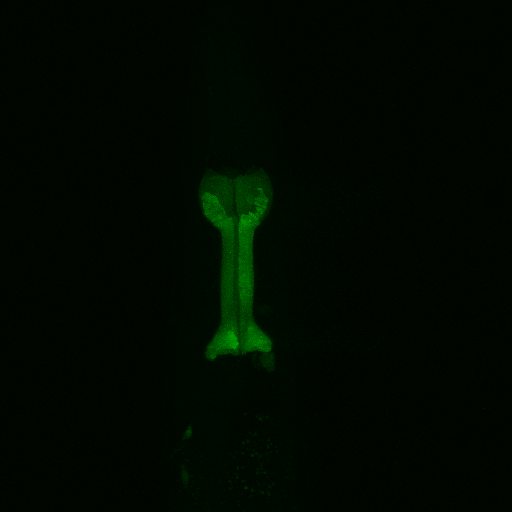

Supplement: Source data 1. [file elife-103718-data1.zip › Figure 4-source data/Figure 4-source data 1(A)/1-WT.png]

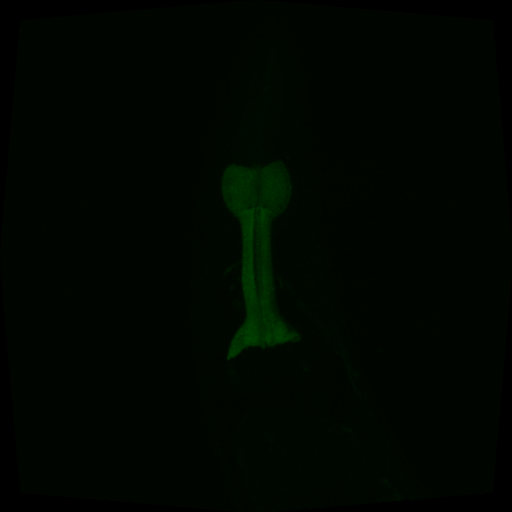

Supplement: Source data 1. [file elife-103718-data1.zip › Figure 4-source data/Figure 4-source data 1(A)/2-ubr-1.png]

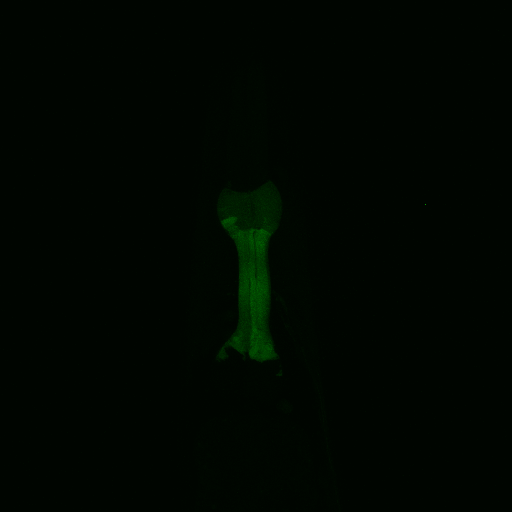

Supplement: Source data 1. [file elife-103718-data1.zip › Figure 4-source data/Figure 4-source data 1(A)/3-+UBR-1.png]

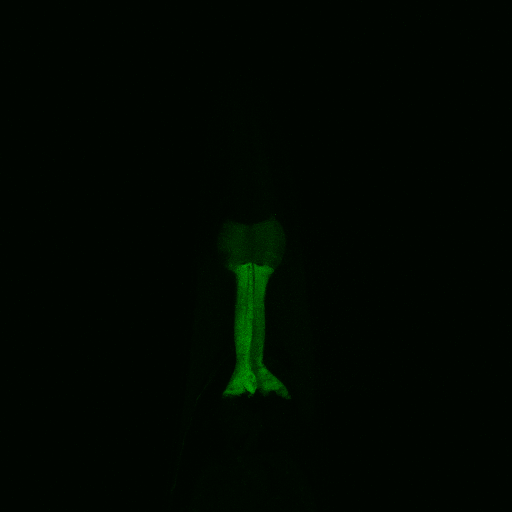

Supplement: Source data 1. [file elife-103718-data1.zip › Figure 4-source data/Figure 4-source data 1(A)/4-ubr-1;got-1.png]

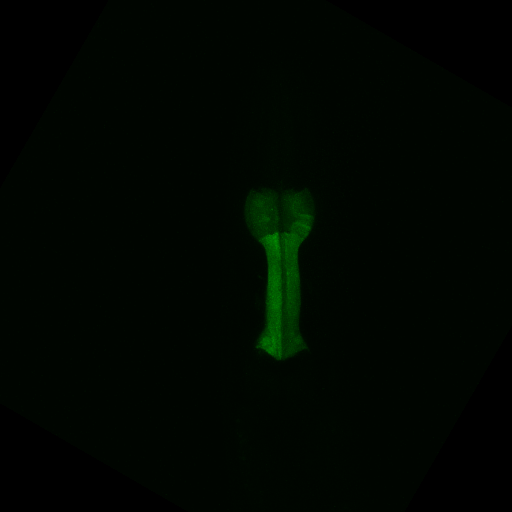

Supplement: Source data 1. [file elife-103718-data1.zip › Figure 4-source data/Figure 4-source data 1(A)/5-ubr-1;eat-4.png]

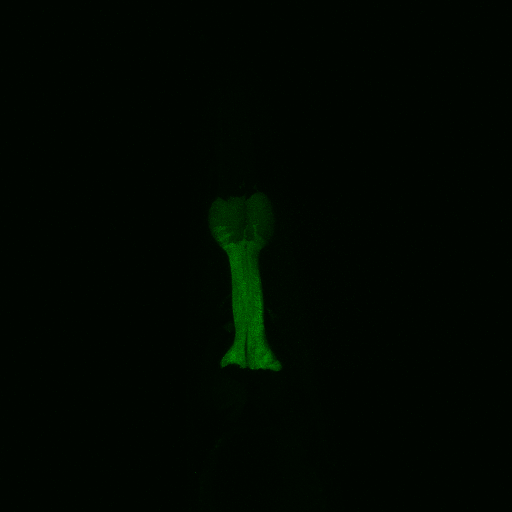

Supplement: Source data 1. [file elife-103718-data1.zip › Figure 4-source data/Figure 4-source data 1(A)/6-ubr-1+Cef.png]

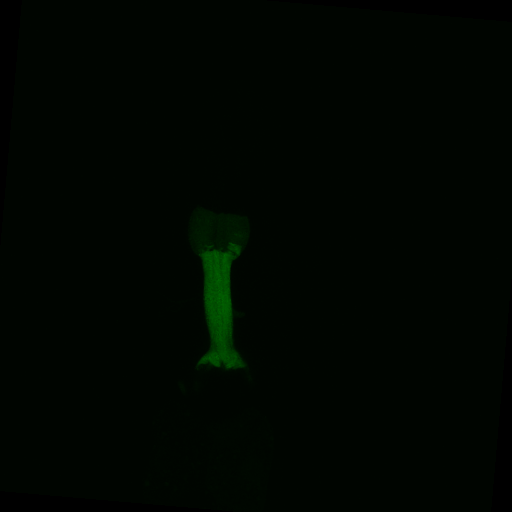

Supplement: Source data 1. [file elife-103718-data1.zip › Figure 4-source data/Figure 4-source data 1(A)/7-WT+Glu.png]
